# Measles antibody seropositivity among children with allergic diseases: A cross-sectional study in the Japan Environment & Children’s Pilot Study

**DOI:** 10.1371/journal.pone.0257721

**Published:** 2021-09-22

**Authors:** Mayako Saito-Abe, Kiwako Yamamoto-Hanada, Kensuke Shoji, Miori Sato, Makoto Irahara, Yu Taniguchi, Makiko Sekiyama, Nathan Mise, Akihiko Ikegami, Masayuki Shimono, Reiko Suga, Masafumi Sanefuji, Shouichi Ohga, Masako Oda, Hiroshi Mitsubuchi, Isao Miyairi, Yukihiro Ohya

**Affiliations:** 1 Medical Support Center for the Japan Environment and Children’s Study, National Center for Child Health and Development, Tokyo, Japan; 2 Division of Infectious Diseases, National Center for Child Health and Development, Tokyo, Japan; 3 Japan Environment and Children’s Study Programme Office, National Institute for Environmental Studies, Ibaraki, Japan; 4 Department of Environmental and Preventive Medicine, Jichi Medical University, Tochigi, Japan; 5 Regional Center for Pilot Study of Japan Environment and Children’s Study, School of Medicine, University of Occupational and Environmental Health, Fukuoka, Japan; 6 Research Center for Environment and Developmental Medical Sciences, Kyushu University, Fukuoka, Japan; 7 Department of Pediatrics, Graduate School of Medical Sciences, Kyushu University, Fukuoka, Japan; 8 Department of Public Health, Faculty of Life Sciences, Kumamoto University, Kumamoto, Japan; 9 Department of Neonatology, Kumamoto University Hospital, Kumamoto, Japan; Public Health England, UNITED KINGDOM

## Abstract

**Background:**

The relationship between allergic individuals and their responsiveness to routine vaccines has rarely been investigated. This study examined whether the seroprevalence of measles antibody differed between children with and without allergic diseases in the general pediatric population.

**Methods:**

The cross-sectional study was performed within a prospective general birth cohort (a pilot study of the Japan Environment & Children’s Pilot Study [JECS]) of children aged 8 years. The clinical history of allergic diseases, measles, and the concentration of measles immunoglobulin G titers in serum enzyme immunoassay were examined. Fisher’s exact tests were used to assess the relationships between the allergic characteristics of the children and their measles antibody positivity rates.

**Results:**

This study included 162 children. Any allergic disease was reported in 75 (46.3%). The measles antibody positivity rate was 94.7% among children with any allergic diseases and 92.0% among children without allergic diseases. Our results revealed no differences in measles antibody seropositivity between children with allergies and controls.

**Conclusions:**

Children with allergies mount and maintain a comparable immune response to the measles vaccine.

## Introduction

Allergic diseases are characterized by a hypersensitivity response to allergens due to a general shift toward Th2 responses, for which patients with these conditions are considered to have altered responsiveness to routine vaccines [1]. However, the relationship between allergic individuals and their responsiveness to routine vaccines has rarely been investigated. The pilot of the Japan Environment and Children’s Study (JECS) [2, 3] planned to measure measles-specific immunoglobulin G (IgG) antibody titers, which are considered appropriate biomarkers for evaluating immunological responsiveness to routine vaccines. Measles and rubella (MR) vaccinations are routinely administered in the national immunization schedule, under which every child receives two doses of MR vaccine at 1 year to <2 years and 5 years to <7 years of age. There has been no large-scale epidemic in Japan for nearly 10 years, and the effects of natural exposure are extremely low [4].

This study sought to determine whether the response to vaccination differed between allergic and non-allergic children in a general birth cohort (the pilot of the JECS) in Japan.

## Methods

**Fig 1:**
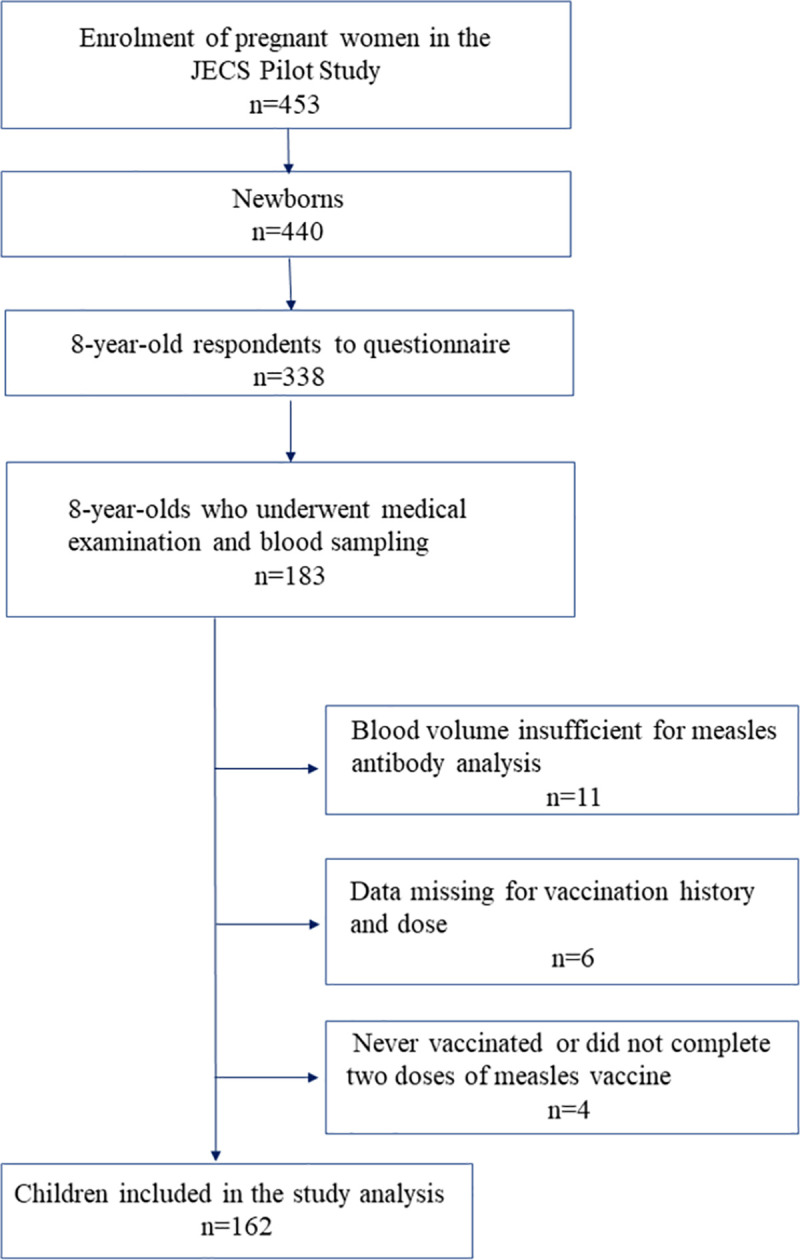
Flow diagram of the patient selection process.

The cross-sectional study design analyzed a prospective general birth cohort, a pilot birth cohort from the JECS. A flow diagram of the patient selection process is shown in Fig 1.

The JECS Pilot Study began 4 years prior to the main JECS to examine the feasibility of various examinations. The JECS Pilot Study was conducted at four regional centers (Jichi Medical University, University of Kyusyu, University of Occupational and Environmental Health, and University of Kumamoto). Between February 2009 and March 2010, a pilot study enrolled pregnant women receiving outpatient care from cooperating local healthcare providers of the regional centers participating in the JECS Pilot Study. A total of 453 pregnant women (participating mothers) agreed to participate. Subsequently, 440 children born to 436 participating mothers were enrolled in the pilot study.

The pilot study consisted of two parts: a questionnaire survey administered to all participants by mail, and a medical examination survey including blood tests and developmental tests, in which the participants visited the regional centers. The medical examination survey was designed so all participants in the pilot study were recruited from each unit, although not all agreed to participate. Of the 440 participating infants, 183 for whom participation consent was provided for the medical examination survey (conducted of those 7–8 years old from December 2019 to December 2020) and blood sampling were included in the analysis. Among them, children for whom insufficient blood samples were available or data were missing regarding vaccination history and doses as well as those who did not receive the two doses of the measles vaccine on schedule were excluded from the study.

Data on demographic factors, history of physician-diagnosed measles, measles vaccination and doses, allergic diseases (asthma, allergic conjunctivitis, allergic rhinitis, and food allergy) were obtained using a questionnaire provided to the caregivers. Atopic dermatitis (AD) was diagnosed using the diagnostic criteria of the UK Working Party. We defined ‘any allergic disease’ as any one of the allergic diseases above. The Patient-Oriented Eczema Measure (POEM) [5], which is used to indicate the severity of AD by patients or parents, was collected at the same time. We divided the POEM score into two categories: normal to almost normal (2 points or less) and mild or more (3 points or more). The total immunoglobulin E (IgE) levels in serum were measured by Immuno-CAP (Thermo Fisher Scientific Inc., Uppsala, Sweden) in independent laboratories, with a high total IgE level of 170 IU/mL or more [6]. Measles IgG titers were also measured using an enzyme immunoassay (EIA) (performed with commercial virus-specific IgG EIA kits [SEIKEN Measles IgG-EIA, Denka Co., Ltd.,Tokyo,Japan]) in independent laboratories. In Japan, the threshold values for a concentration of positive IgG-EIA were ≥4 IU/mL as evidence of a protective antibody for the general public and ≥16 IU/mL for protection from subclinical infection required by healthcare professionals on the basis of previous studies that showed that the antibody concentration for prevention in 98% or more of people was 125–200 mIU/mL, while the subclinical reinfection prevention level was 500–1,000 mIU/mL [7–10]. To convert these thresholds to the international standard unit (mIU/mL), each is multiplied by 45 (company data, Denka Co., Ltd.). Therefore, we set the threshold values in the international standard in the current study to 180 and 720 mIU/mL, respectively.

Fisher’s exact tests were used to assess the relationships between the allergic characteristics of the children and their measles antibody positivity rates. The statistical analyses were performed using JMP*®* 15.1.1. This study was conducted with the approval of the Ethics Committee of the National Institute for Environmental Studies, where the Program Office of JECS is located, and all facilities where the regional unit center is located, in compliance with the ethical guidelines for medical research in humans. Written informed consent was obtained from all caregivers of the participants before the participants’ inclusion in the study.

## Results

**Fig 2:**
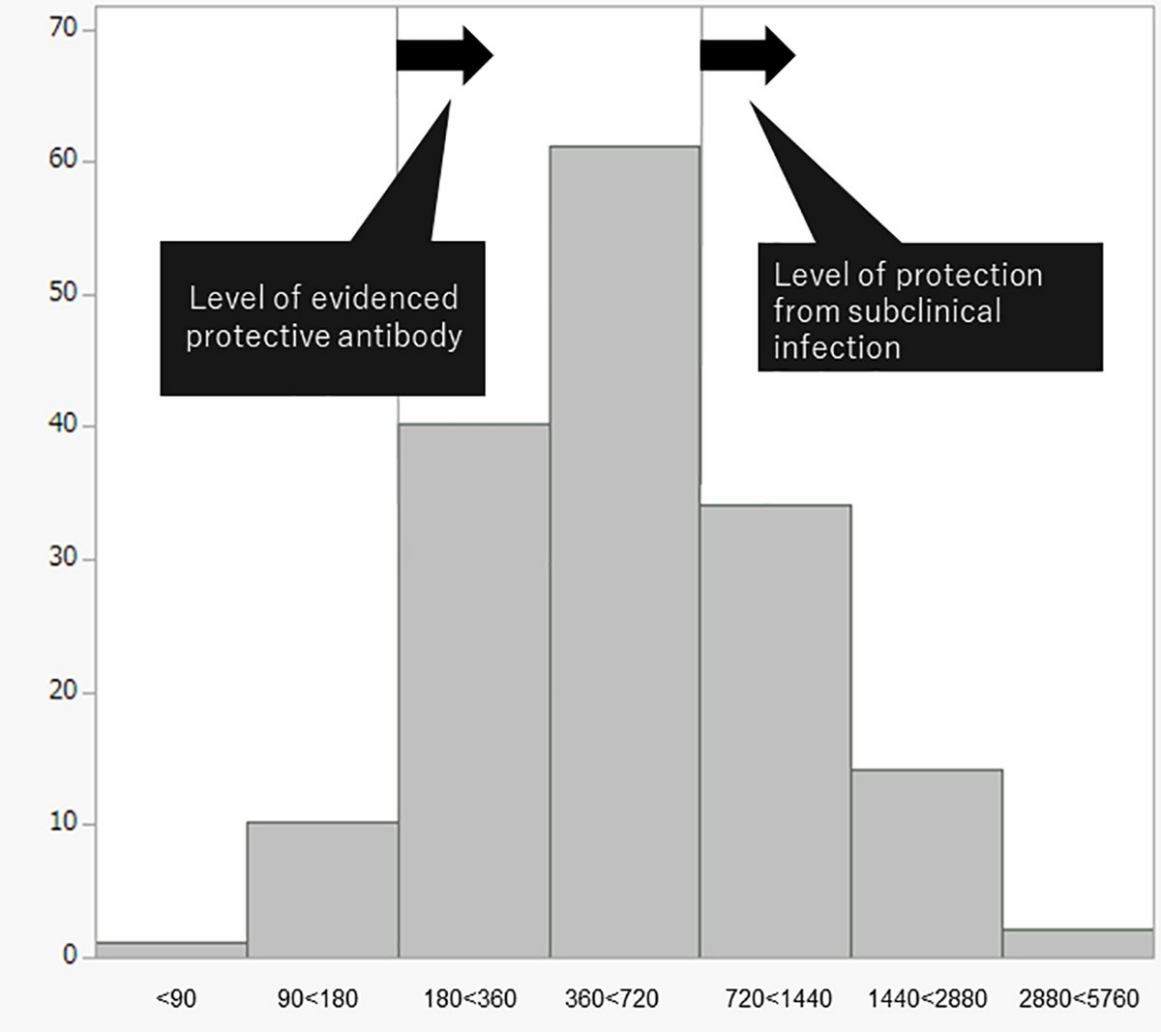
The distribution of measles antibody titers of the subjects (n = 162). The X-and Y-axes show the measles antibody (mIU/mL) and the number of participants, respectively.

The study included 162 children. Fig 2 shows the distribution of measles antibody titers (n = 162). The median measles IgG titer was 508.5 mIU/mL (interquartile range [IQR], 326.3–803.3 mIU/mL).

A positive response to the measles vaccine corresponding to the evidenced protective antibody level was observed in 151 (93.2%) children, while a positive response to the measles vaccine for the prevention of subclinical infection was observed in 50 (30.9%) children.

Table 1 shows the baseline characteristics of the children and the associations between them and their positive responses to the measles vaccine. None of the children had a history of measles infection. High total IgE (170 IU/mL or more) was found in 45.1%. Allergic diseases were common among the children (any allergic diseases, 46.3%; asthma, 19.7%; allergic conjunctivitis, 12.2%; allergic rhinitis, 31.5%; food allergy, 14.6%; and atopic dermatitis, 27.8%). The measles antibody positivity rate was 94.7% among children with allergic diseases and 92.0% among children without allergic diseases for the evidenced protective antibody level, showing no significant difference. The median measles IgG titer and interquartile range (IQR) among children with versus without a history of allergic diseases was 459 (IQR, 311–801) and 522 (IQR, 350–808), respectively (data not shown). Sex, history of each allergic disease, severity of AD by POEM, and current treatment of asthma/AD also did not show significant differences in the positive responses to the measles vaccine. Since none of the children in this study were obese (represented by a body mass index of 25 or higher), the relationship between obesity and antibody positivity could not be examined.

**Table 1:** The allergic characteristics of the participants and their measles antibody positivity rates.

			The evidenced protective antibody level			The prevention level of subclinical infection^§^		
		All participants n (%)	Positive	Negative	p value*	Positive	Negative	p value*
			n (%)	n (%)		n (%)	n (%)	
All participants		-	151 (93.2)	11	-	50 (30.9)	112 (69.1)	-
(n^†^ = 162)				(6.8)				
Sex	Male	83 (51.6)	78 (48.5)	5 (3.1)	0.76	20 (12.4)	63 (39.1)	0.08
(n^†^ = 162)	Female	78 (48.5)	72 (44.7)	6 (3.7)		29 (18.0)	49 (30.4)	
Body mass index (n^†^ = 161)	≥25	0 (0.0)	-	-	-	-	-	-
	<25	161 (100.0)						
Clinical history of measles		0 (0.0)	0 (0.0)	0 (0.0)	-	0 (0.0)	0 (0.0)	-
(n^†^ = 162)								
Total IgE (IU/mL)	≥170	73(45.1)	69 (42.6)	4 (2.5)	0.76	26 (16.1)	48 (29.0)	0.31
	<170	89 (54.9)	82 (50.6)	7 (4.3)		24 (14.8)	65 (40.1)	
(n^†^ = 162)								
Any allergic diseases‡	Yes	75 (46.3)	71 (43.8)	4 (2.5)	0.55	21 (12.9)	54 (33.3)	0.50
(n^†^ = 162)	No	87(53.7)	80 (49.4)	7 (4.3)		29	58 (35.8)	
						(17.9)		
							22 (14.7)	
Asthma	Yes	29 (19.3)	28 (19.9)	1 (0.7)	1.00	7 (4.7)		0.50
(n^†^ = 150)	No	121 (80.7)	113 (75.3)	8 (5.3)		39(26.0)	82 (54.7)	
Current treatment	Yes	6 (3.8)	5 (3.1)	1 (0.6)	0.35	2 (1.3)	4 (29.4)	1.00
	No	154 (96.3)	144 (90.0)	10 (6.3)		47 (29.4)	107 (66.9)	
(n^†^ = 160)								
Allergic conjunctivitis (n^†^ = 147)	Yes	18 (12.2)	17 (11.6)	1 (0.7)	1.00	6(4.1)	12 (8.2)	0.79
	No	129 (87.8)	121 (82.3)	8 (5.4)		40 (27.2)	89 (60.5)	
Allergic rhinitis	Yes	47	44 (29.5)	3	1.00	13	34	0.57
		(31.5)		(2.0)		(8.7)	(22.8)	
(n^†^ = 149)	No	102 (68.5)	95 (63.8)	7 (4.7)		34 (22.8)	68 (45.6)	
Food allergy	Yes	22 (14.6)	21 (13.9)	1 (0.7)	1.00	6 (4.0)	16 (10.6)	1.00
(n^†^ = 151)	No	129 (85.4)	121 (80.1)	8 (5.3)		39 (25.8)	90 (59.6)	
Atopic dermatitis	Yes	45 (27.8)	43 (26.5)	2 (1.2)	0.73	14 (8.6)	31 (19.1)	1.00
(n^†^ = 162)	No	117 (72.2)	108 (66.7)	9 (5.6)		36 (22.2)	81 (50.0)	
Current treatment	Yes	16 (10.0)	14 (8.8)	2 (1.3)	0.27	4 (2.5)	12 (7.6)	0.78
	No	143 (89.9)	135 (84.9)	8 (5.0)		44 (27.7)	99 (62.3)	
(n^†^ = 159)								
POEM^‡^	mild or more	31 (19.1)	27 (16.7)	4 (2.5)	0.22	9(5.6)	22 (13.6)	1.00
(n^†^ = 162)	normal to almost normal	131 (80.9)	124 (76.5)	7 (4.3)		41 (25.3)	90	
							(55.6)	

^¶^ Measles antibody of180mIU/mL or higher.

^§^ Measles antibody of 720mIU/mL or higher.

s Fisher’s exact test.

^†^ Number of participants without missing values.

^‡^ Severity of atopic dermatitis, Patient-Oriented Eczema Measure.

‡ Defined as any one of allergic disease.

## Discussion

The results of the current study revealed that the responses to measles vaccination did not differ between allergic and non-allergic children in a general birth cohort in Japan. The strength of this study was that our study design was structured in the general population, minimizing sampling bias.

Juhn *et al*. reported that measles IgG titers did not differ between asthma and non-asthmatic patients in a retrospective medical chart review in the US [11]. Our study results were consistent with those of this previous study, in that the responsiveness to measles vaccine was similar between allergic and non-allergic individuals based on the evaluation of asthma and other allergic diseases. However, previous studies suggested that responses to non-measles vaccines may differ by the presence or absence of allergic disease. Sheen et al. reported that poorer vaccination responses to the 23-valent pneumococcal polysaccharide vaccine (PPSV-23) were observed in asthmatic individuals compared to controls [12]. Paulke-Korinek et al. reported that when adults were administered a booster dose of the tick-borne encephalitis (TBE) vaccine, allergic individuals had higher anti-TBE titers than non-allergic individuals [13]. We speculated that the immunological response to the pathogens might differ in the type of organisms in allergic and asthmatic children. Further studies are needed to elucidate the mechanisms of the immune response to various pathogens in allergic children.

Some limitations should be considered when interpreting our results. First, 54.1% of our targeted participants who completed the questionnaire at 8 years of age underwent blood testing in this study. All participants were guided through a medical examination survey including blood sampling, but only those who could meet the proposed schedule could participate. However, our participants were considered suitable for testing the research hypothesis since the distribution of those with and without allergic disease was approximately even. The total number of children was sufficient compared to previous reports that tested the same kind of hypothesis. Second, this study did not determine the severity of allergic diseases other than AD. Since the current study was conducted of a general birth cohort, the majority of cases of AD due to POEM were mild. There is room for further study regarding the acquisition and maintenance of antibodies against vaccines in children with severe allergic diseases. Third, we did not have information about allergic disease treatment at the time of the measles immunization. It was suggested that the effect of maintenance corticosteroid therapy, especially systemic steroids and high-dose inhaled corticosteroids, may affect the immune response to vaccinations among asthma patients [14]. As a reference, according to the data at the time of the blood sampling, only 6 children were taking any medication for asthma and none were on systemic steroids. The possibility of immunosuppressive steroid use at the time of the immunization seemed very low, and it was considered not to have a significant impact on our results.

## Conclusions

In conclusion, the results of this study showed no differences in the positive responses to measles vaccine between children with and without allergic diseases in Japan. These results could reassure and provide information regarding measles prevention after measles vaccinations in children with allergic diseases.
